# Sleep Characteristics in Diabetic Patients Depending on the Occurrence of Neuropathic Pain and Related Factors

**DOI:** 10.3390/ijerph17218125

**Published:** 2020-11-03

**Authors:** Cristina Naranjo, María Dueñas, Carlos Barrera, Guillermo Moratalla, Inmaculada Failde

**Affiliations:** 1 University Hospital Puerta del Mar, Avda. Ana de Viya 21, 1009 Cádiz, Spain; cristina.naranjo.munoz@gmail.com (C.N.); carlosbarreratenorio@gmail.com (C.B.); 2 Department of Statistics and Operational Research, University of Cadiz, Calle Enrique Villegas Vélez, 2, 11002 Cádiz, Spain; 3 Biomedical Research and Innovation Institute of Cádiz (INiBICA), Avda. Ana de Viya 21, 11009 Cádiz, Spain; inmaculada.failde@uca.es; 4 The Observatory of Pain (External Chair of Pain), Grünenthal Foundation, University of Cádiz, Avda. Ana de Viya 52, 11009 Cádiz, Spain; 5 Primary Care Center Loreto-Puntales, Health district Bahía de Cádiz-La Janda, C/ Hidroavión Numancia 0, 11011 Cádiz, Spain; morrogui@gmail.com; 6 Preventive Medicine and Public Health Area, University of Cádiz, Avda. Ana de Viya 52, 11009 Cádiz, Spain

**Keywords:** diabetes mellitus type 2, diabetes neuropathic pain, sleep disorders, sensorial phenotype, anxiety, depression

## Abstract

This study aims to compare the sleep characteristics (structure and quality) in patients with type-2 diabetes mellitus with and without diabetic neuropathic pain (DNP), and to investigate the relationship of sensory phenotypes, anxiety, and depression with sleep quality in DNP patients. A cross-sectional study was performed in patients with type-2 diabetes mellitus and neuropathy. Patients were classified into two groups—with or without neuropathic pain—according to the “Douleur Neuropathique-4 (DN4)” scale. Sleep characteristics and quality (Medical Outcomes Study—MOS-sleep), pain phenotype (Neuropathic Pain Symptom Inventory—NPSI), mood status (Hospital Anxiety and Depression scale—HADS), pain intensity (Visual Analogue Scale—VAS), and quality of life (SF-12v2) were measured. The sample included 130 patients (65 with DNP). The mean scores in all the dimensions of the MOS-sleep scale were higher (more disturbances) in the DNP patients. Higher scores in anxiety or depression, greater intensity of pain or a higher score in the paroxysmal pain phenotype were associated with lower sleep quality in DNP patients. A shorter duration of the diabetes and lower levels of glycated hemoglobin were also associated with lower sleep quality. The results show the relationship between DNP and sleep quality, and the importance of assessing sensory phenotypes and mental comorbidities in these patients. Taking these factors into consideration, to adopt a multimodal approach is necessary to achieve better clinical results.

## 1. Introduction

Sleep is a basic physiological requirement for humans that has been shown to play a part in the correct functioning of memory, emotions, and learning [1]. By the same token, sleep disorders (SD) have been shown to affect around 20–30% of the western population on a daily or weekly basis, leading to a lower quality of life [1]. These disorders have also been reported to be a risk factor for diseases such as diabetes, and they can encourage the presence of diabetes complications [1,2,3,4]. In fact, between 42–77% [3] of patients with type-2 diabetes mellitus (DM-2) are reported to suffer from sleep disorders, although few studies have analyzed their relationship with patient outcomes and quality of life [5].

Several studies have shown that between 50–90% of people with chronic pain also suffer from some kind of SD—insomnia being the most common [1,6,7]—while other studies have established a two-directional relationship between the two processes [7,8,9], with clinical and neurobiological evidence supporting this association [1,6,10]. Consequently, the study of SD has become a subject of particular interest regarding patients with pain conditions as it could help to improve the results of their treatment [10].

In this vein, several studies have found a relationship between SD and neuropathic pain in patients with DM-2, highlighting the need to include detailed assessments of pain when exploring the SD of these patients [3]. The analysis of sensory phenotypes is a more frequently recommended strategy in patients with neuropathic pain because different phenotypes have been shown to reveal different neurobiological pain mechanisms [11,12], which could explain the heterogeneity of the patients [12] and the variability in clinical outcomes [11,13,14].

Finally, it is worth highlighting that negative mood and anxiety are frequently observed processes in diabetic patients [15] and patients with neuropathic pain of different origins [16], and that the management and outcome of neuropathic pain in diabetic patients can worsen when these processes coexist with SD [2,17,18].

Due to the potential relevance of SD in patients with DM-2, this study aims to compare the sleep characteristics (quality and structure) in all their dimensions in patients with DM-2 with and without diabetic neuropathic pain (DNP), and as a second objective, to investigate the relationship of anxiety, depression, and the sensory phenotypes of pain with sleep quality in DNP patients.

## 2. Materials and Methods

### 2.1. Participants

A cross-sectional study was carried out between June 2017 and July 2018 in 4 primary health care centers in Cádiz (Spain). The patients participating were adults diagnosed with DM-2, based on the criteria of the American Diabetes Association (ADA), and others with diabetic neuropathy, based on a clinical examination and the monofilament test [19].

All the participants had to be mentally and physically able to answer the questionnaire and to provide their written informed consent in advance. The study was approved by the Clinical Research Ethics Committee of the University Hospital “Puerta del Mar” (Cádiz, Spain) (Reference Number of the study: 36/17), ensuring compliance with the standards of good clinical practice, and it was performed in accordance with the Helsinki Declaration.

### 2.2. Selection Process

Patients were selected from the “Diabetes Mellitus Integrated Care Process”, registered in an electronic unique health record system for primary care management (DIRAYA System, Andalusian Health Service, Andalusia, Spain). Approximately 2000 DM-2 patients were initially identified from this system. Then, the selection of the individuals with diabetic neuropathy was based on searching for patients at risk who presented at least one of three indicators of poor control of the diabetes mellitus: a glycated hemoglobin (HbA1c) level over 8%; a diagnosis of diabetic retinopathy or foot ulcers; and having been first diagnosed with the illness more than 10 years earlier. The patients selected were contacted, had the objectives and characteristics of the study explained to them, and were invited to participate. The patients who agreed to participate attended a meeting at the primary care center, where they were informed about the right to withdraw from the interview at any time and the need for their informed consent.

A well-trained member of the research group conducted the clinical interview and performed the foot examination with the monofilament test according to the standard procedure. Patients with a negative examination, presenting mental impairment, or who were unable to complete the questionnaire were excluded from the study. Finally, a sample of 130 patients with DM-2 and neuropathy was obtained.

These patients were classified into two groups, with or without neuropathic pain (case and controls respectively) depending on the results in the “Douleur Neuropathique 4 questions (DN4)” scale [20]. This instrument has been adapted and validated in the Spanish language and consists of 10 binary items with a summary index measure that distinguishes between subjects with DNP (score over or equal to 4) or without. The Spanish version of the instrument presents a sensibility of 79.8% and specificity of 78.0% [20].

The sociodemographic, clinical, and sleep characteristics (structure and quality) of both groups were described. Furthermore, the factors related to sleep quality were analyzed in the patients with neuropathic pain (DNP).

### 2.3. Instruments and Variables

Information was collected from each patient’s medical records and from a structured questionnaire.

The Medical Outcomes Study (MOS) Sleep Scale was used to assess sleep characteristics (structure and quality). This is a validated scale in Spanish with appropriate psychometric properties to assess the sleep characteristics of patients with chronic pain (including neuropathic pain) with good reliability, validity, and sensitivity to change [21,22]. This instrument consists of 12 items that examine the impact of the disease on the dimensions and patterns of sleep, i.e., the structure of the sleep; sleep disturbances (including the items: having trouble falling asleep—item 7; how long to fall asleep—item 1; sleep not being quiet—item 3; awakening during your sleep time—item 8; snoring—item 10; having shortness of breath or headache—item 5; sleep adequacy—get enough sleep to feel rested upon waking in the morning—item 4; getting the amount of sleep needed—item 12; daytime somnolence—drowsy during day—item 6; having trouble staying awake during the day—item 9; and taking naps—item 11). In addition, sleep quality is measured by a summary index (the sleep problem index “I-9”), which is constructed from items 1,3,4,5,6,7,8, and 9 and provides complete information about sleep quality. Each dimension is scored independently, ranging from 0–100, a higher score indicating more sleep problems. The quantity of sleep is scored as the average number of hours slept per night during the last 4 weeks, where 7–8 h of sleep is considered optimal.

Furthermore, data were gathered on sociodemographic variables (age, sex, educational level, and employment status), and on clinical variables such as time since diagnosis of DM-2, duration of DNP, treatment with insulin, presence of cardiovascular risk factors (obesity, arterial hypertension, and dyslipidemia), last level of glycated hemoglobin (HbA1c) recorded, pharmacological treatment for sleep or for pain relief, and presence of DM-2 complications (retinopathy, nephropathy, diabetic foot, and cardiovascular disease). Data on physical comorbidities were also collected. A patient was considered to have physical comorbidities if they had any other pathology in their medical record other than the diabetes complications, cardiovascular risk factors, mood disorders, or sleep problems previously considered.

The neuropathic pain phenotype was identified using the Neuropathic Pain Symptom Inventory (NPSI). This scale, which is also adapted and validated in Spanish, presents different values of sensitivity and specificity depending on the criterion used. First, the authors propose a clinical criterion: a reduction of at least 30% of the NPS total score from the first to the second assessment and/or an absolute two-point reduction in the first item. With this criterion, a sensitivity of 68.0% and a specificity of 87.2% were observed. A second criterion (discriminant criterion) is based on the score of the discriminant function of the Neuropathic Pain Questionnaire-Short Form (NPQ-SF). This score is used to classify patients with and without a relevant neuropathic component at the second assessment. With this criterion, a sensitivity of 85.0% and a specificity of 75.2% were found. This scale includes 12 items: 10 descriptors allow us to quantify the five most relevant clinical dimensions of the neuropathic pain syndrome scored with a range of 0–10 [23]. The dimensions included are: evoked pain, deep spontaneous pain, superficial spontaneous pain, paroxysmal pain, and paresthesia/dysesthesia.

Pain intensity was measured using a visual analogue scale (VAS) with a range of 0–10, with 0 corresponding to no pain and 10 to the worst pain possible.

The Hospital Anxiety and Depression scale (HAD) [24] was used to assess mood status (anxiety and depression). It is a valid and reliable instrument recommended for assessing the emotional status of patients with painful diabetic neuropathy [25]. The HAD is a simple measure that consists of two seven-item subscales: HAD-A for anxiety and HAD-D for depression. Each item is scored on a four-point Likert scale and the subscale scores range from 0–21. A score above 10 indicates clinically significant anxiety or depression [26]. Two binary variables were created according to this threshold to assess the presence of anxiety and depression. It has been shown that the HAD-A and HAD-D scales possess satisfactory psychometric properties for detecting the presence of anxiety and depression in the Spanish population [27], showing a sensitivity above 60% and a specificity above 70% in most studies [28].

Quality of life (QoL) was measured using the SF-12 v2 Health Survey [29]. This instrument contains 12 items that make it possible to calculate the profile of 8 dimensions: physical functioning (PF), role-physical (RP), bodily pain (BP), general health perception (GH), vitality (V), social functioning (SF), role-emotional (RE), and mental health (MH). In addition, two global scores arise from those 8 domains: the physical health component summary (PSC-12) and mental health component summary (MSC-12). Each of the 12 items is measured by a Likert-type response of 3 or 5 elements and all range from 0–100, with higher scores referring a better state.

### 2.4. Statistical Analysis

A descriptive analysis was performed in both groups of patients (with and without neuropathic pain) using measures of frequency, central tendency, and dispersion. In addition, to analyze the associations between the variables studied (sociodemographic and clinical) and the two groups of patients (with and without neuropathic pain), and to compare the structure and quality of sleep in both groups and the associations of the Sleep Problem Index (I-9) of the MOS sleep scale with the variables studied, chi-squared tests, *t*-tests, ANOVA, Mann–Whitney U, Kruskal Wallis, and correlation coefficients (Pearson or Spearman) were performed, depending on the type and on the distribution of the variables, assessed by the Kolgomorov–Smirnov test. Furthermore, Pearson or Spearman coefficients were calculated to analyze the correlations between each dimension of the MOS-Sleep Scale and each dimension of the NPSI, in the diabetic patients with neuropathic pain.

Finally, in the patients with DNP, a multivariate linear regression model was performed to analyze the variables related to sleep quality (measured with the I-9), where the dependent variable was the Sleep Problem Index (I-9) of the MOS sleep scale. Included in the models were the significant variables (*p* < 0.05) identified in the bivariate analysis and those considered relevant according to the scientific literature. Tolerance and the variance inflation factor (VIF) were also computed. We assumed that collinearity was not present when the VIF value was below 5 and the tolerance score over 0.2 [30,31].

These analyses were all carried out with the IBM SPSS Statistics 24^®^ package (IBM Corporation, Armonk, NY, USA).

## 3. Results

### 3.1. Characteristics of the Sample

Of the 130 individuals included in this study, 65 had DNP. As observed in Table 1, the percentage of women was higher among the patients with DNP (58.5% vs. 44.6%). In addition, the DNP patients were also younger (70.3 vs. 74.8) and unemployed or homemakers (43.1% vs. 32.3%) (Table 1).

Table 1 also shows that the evolution time of the diabetes averaged approximately 12 years in both groups and the presence of complications was similar, cardiovascular disease being the most common complication in both groups (46.2% and 43.1%, respectively). Besides, the patients with DNP were more frequently obese (63.1% vs 38.5%) and had dyslipidemia (80% vs 63.1%), although arterial hypertension was more common in the subjects without DNP (81.5% vs 73.8%). Physical comorbidities were high (90.8%) in the patients with DNP, and also in those without (Table 1).

The patients with DNP took sleep medication with greater frequency (55.4% vs. 30.8%) for pain relief (76.9% vs. 52.3%) and were under treatment with insulin (69.2% vs. 50.8%) (Table 1). Likewise, the scores in the scales for anxiety (9.3 vs 4.3) and depression (8.7 vs. 4.8) were higher among the patients with DNP. In both the physical (30.4 vs. 38.9) and mental (42.1 vs. 52.9) component of the SF-12, the scores were lower among the group of patients with DNP (Table 1).

In the DNP patients, the pain duration was 4.8 years (SD = 3.4) on average and the pain intensity was 7.48 (SD = 2.2). In the NPSI dimensions the highest scores were paresthesia/dysesthesia (5.43 SD = 2.75), followed by paroxysmal pain (4.85 SD = 2.95) and superficial spontaneous pain (4.26 SD = 3.95). The score in evoked pain (3.23 SD = 2.58) and deep spontaneous pain (3.23 SD = 2.58) were lower.

### 3.2. Sleep Characteristics as Defined by the MOS Sleep Scale

As shown in Table 2, the mean scores observed in the Sleep Problem Index (I-9) and in all the dimensions of the MOS sleep scale were higher (more disturbances) in the DNP patients than in the control subjects. Moreover, the proportion of painless patients with optimal sleep was higher than that observed in the pain group (Table 2).

As Table 3 shows, evoked pain had the highest correlation with the dimensions of the MOS sleep scale. That is, the higher the score in the evoked pain dimension of the NPSI, the higher the scores on the dimensions that explore sleep structure. An exception was the number of hours of sleep, which was lower in patients with more evoked pain. Paroxysmal pain was related with sleep adequacy (*r* = 0.305) and the Sleep Problem Index (I-9) (*r* = 0.277), while deep spontaneous pain and paresthesia/dysesthesia were significantly related with the short of breath or headache dimensions (*r* = 0.293 and *r* = 0.269, respectively). In all cases, a higher score in the dimension of sensory phenotype was associated with greater sleep problems (Table 3).

### 3.3. Variables Associated with Sleep Quality (Sleep Problem Index) in the Patients with DNP

Table 4 shows that among the whole sample of DNP patients, it was women, younger patients and those with a lower educational level who had the highest scores in the Sleep Problem Index (I-9) of the MOS scale, i.e., worse sleep quality. In addition, as the intensity of pain increased, the duration of diabetes decreased, and the level of glycated hemoglobin (HbA1c) was lower, the score on the Sleep Problem Index (I-9) increased, i.e., worse sleep quality (Table 4).

No significant relationship was found between sleep quality and complications of DM-2 or cardiovascular risk factors, although, in general, the patients presenting these problems had a higher score in the Sleep Problem Index (I-9) (Table 4).

Worse sleep quality was observed in the patients with a higher score on the HADS-D and HADS-A scale (depression and anxiety), as well as among those taking medication for sleep (Table 4). With regard to HRQL, we observed worse sleep quality when the score in the mental component of quality of life was lower. However, no differences were detected in the physical component.

### 3.4. Multivariate Analysis of the Variables Associated with the Sleep Problem Index (I-9) in DNP Patients

Table 5 shows two adjusted models of the variables associated with sleep quality, measured by the Sleep Problem Index (I-9). They differ in that model 1 includes pain intensity as the independent variable, while model 2 includes paroxysmal pain. The decision was made to include these variables in two separate models because of the potential collinearity between them, and the hypothesis that the sensory phenotypes could have a different effect on the Sleep Problem Index (I-9) than pain intensity. The models show that, in the sample of DNP patients, higher scores on the anxiety or depression scales and a greater intensity of pain (model 1) or greater score in the paroxysmal pain phenotype (model 2) were associated with a higher score in the Sleep Problem Index (I-9), i.e., worse sleep quality (Table 5). On the other hand, a shorter duration of the diabetes and a lower level of glycated hemoglobin (HbA1c) were associated with worse sleep quality (higher Sleep Problem Index (I-9)).

The variables indicating that patients were taking medication for sleep and for pain relief were initially included in the models as they were possible confounding variables. However, they were found not to be significant and did not alter the coefficients of the remaining variables in the models so they were removed from the final models.

## 4. Discussion

This study analyzes the differences in the characteristics (quality and structure) of sleep in patients with type-2 diabetes with and without neuropathic pain, in addition to the factors associated with sleep quality in DNP patients, including sensory phenotypes.

Of note among the results obtained, the score in the MOS-sleep scale was higher (more sleep disorders) among the patients with DNP than among those not presenting neuropathic pain, and the differences between these groups were observed in all the dimensions of the MOS sleep scale. These results are in agreement with those reported by other authors, such as Zhu B et al. (2018) and Tanik N et al. (2016), who show a relationship between DNP and the presence of sleep disorders in DM-2 patients [3,32], and O’Brien et al. (2010), who highlight the need to assess these disorders taking their dimensions into consideration [33].

Sleep problems have been shown to be an emerging factor associated with a greater risk of diabetes. Systematic reviews and meta-analyses have demonstrated that a low quantity and quality of sleep increases the risk of developing DM-2 [34], and that poor sleep is inversely associated with quality of life [35]. However, few studies have analyzed the relationship between sleep problems and DNP, despite the fact that a greater understanding of this relationship could potentially reduce the risk of this complication and improve the quality of life of diabetic patients.

What is particularly significant in the study is that in the DNP patients, the pain intensity and the evoked pain and paroxysmal pain phenotypes presented positive correlations with different dimensions of the MOS sleep scale (higher scores leading to worse sleep quality), while evoked pain correlated negatively with the quantity of sleep.

These results support the hypothesis that the expression of pain in DNP patients will affect sleep quality in different ways, and are in agreement with those obtained by other authors, who highlight the importance of analyzing sensory phenotypes and their potential therapeutic implications [12,36,37]. Calvo et al. (2019) also emphasize the importance of stratifying patients according to sensory profiles on the basis that certain drugs such as gabapentin or pregabalin improve the latency and depth of sleep in these patients, while duloxetine improves sleep fragmentation [38].

In a review of the mechanisms that may explain the presence of neuropathic pain in diabetic patients, Rosenberger et al. (2020) indicate that lesions or diseases affecting the somatosensory nervous system not only lead to a loss of function, but also to overexcitability and increased sensitivity to painful stimuli (hyperalgesia), pain sensations to normally non-painful stimuli (allodynia) and spontaneous pain [39]. Although signs of sensory gain were traditionally thought to be rare in painful diabetic neuropathy, recent studies using QST have demonstrated a high prevalence of mechanical hyperalgesia in painful diabetic neuropathy [40,41,42].

Another result of the study is that higher scores on the HADS anxiety and depression scales were associated with higher scores in the Sleep Problem Index (I-9) of the MOS-sleep scale (worse quality of sleep). Earlier studies have shown a relationship between chronic pain, depression, and SD, suggesting the existence of neurobiological correlations between these processes [6]. Ojeda et al. (2018) refer to mood disorders possibly increasing or perpetuating the impact of SD on pain, possibly through an increase in physiological or cognitive excitement, or due to a deregulation of daily sleep patterns [16]. However, to confirm this hypothesis, it would be necessary to extend the research to the area of neurobiology, and specifically to DNP patients.

A shorter time evolution of DM-2 was another variable associated with higher scores in the Sleep Problem Index (I-9) of the MOS-sleep scale among the patients in the study. These results could be explained by the fact that sleep quality can be affected by factors related to adaption to diabetes, recently-diagnosed patients being less adapted. This result could also be explained by the presence of other common comorbidities in these patients [43]. However, in the present study, although physical comorbidities were common, data about the specific cause of this comorbidity were not collected.

The patients with better levels of HbA1c also presented higher scores in the Sleep Problem Index (I-9). Although these results may appear to contradict those reported by other authors, they are in agreement with those presented by Zhu et al. (2017) in a review of the relationship between sleep disturbance and glycemic control in adults with DM-2 [4]. They found inconclusive results, in particular with regard to the quantity of sleep, nine out of 28 studies showing no relationship between any measure of sleep and glycemic control.

Finally, it is worth mentioning that the DNP patients were taking more medication for sleep and pain control than those not suffering from pain. However, when these variables were introduced into the models, the coefficients of the other variables remained unchanged, which is why they were excluded.

### Limits and Strengths

Among the limitations of the study, it is important to highlight that the sample was obtained using a consecutive non-random sampling technique based on the selection of high-risk type-2 diabetic patients due to the lack of a record of patients with diabetic neuropathy and DNP. This procedure could justify the small differences observed between the groups.

An additional limitation was that detailed information about the subjects’ treatment was not collected. Furthermore, the cross-sectional design of the study is a limitation in itself, as it does not allow for the analysis of causal relationships between the variables analyzed.

As a strength, we would highlight the inclusion of the NPSI, with proven validity and a high correlation with other measures of patient sensitivity such as QST [44], which has made it possible to determine and analyze the sensory profiles of the patients in the study and the use of validated instruments to collect the information. The study performs an in-depth analysis of SD and associated factors in DNP patients, taking into consideration their sensory profiles, which is an innovative topic that has not been studied before.

## 5. Conclusions

The results obtained show the relationship between DNP patients and the quality of their sleep, and the importance of assessing sensory phenotypes and mental comorbidities in these patients. Taking these factors into consideration supports the belief that it is necessary to adopt a multimodal approach to achieve better clinical results for these patients.

## Figures and Tables

**Table 1:** Characteristics of the patients with and without DNP.

Variables	Categories	Cases (Pain DN4 ≥ 4) *n =* 65	Controls (No pain DN4 ≤ 4) *n =* 65	*p*-Value
		*n* (%)	*n* (%)	
**Sociodemographic data**				
Gender	Men	27 (41.5)	36 (55.4)	0.114 ^a^
	Women	38 (58.5)	29 (44.6)	
Age	Mean (SD)	70.25 (10.01)	74.75 (8.96)	0.009 ^d^
Education level	No education	20 (30.8)	20 (30.8)	0.825 ^b^
	Primary studies	33 (50.8)	33 (50.8)	
	Secondary studies	10 (15.4)	8 (12.3)	
	University studies	2 (3.1)	4 (6.2)	
Employment status	Unemployed	5 (7.7)	2 (3.1)	0.092 ^b^
	Homemaker	23 (35.4)	19 (29.2)	
	Working	3 (4.6)	0 (0)	
	Retired	22 (33.8)	33 (50.8)	
	Partial disability	1 (1.5)	0 (0)	
	Total disability	11 (16.9)	11 (16.9)	
**Clinical data**				
Time since type-2 diabetes mellitus diagnosis (years)	Mean (SD)	11.59 (3.29)	12.32 (2.94)	0.184 ^c^
HbA1c registered	Mean (SD)	7.5 (1.37)	7.41 (1.31)	0.571 ^d^
Medication for sleep	Yes	36 (55.4)	20 (30.8)	0.005 ^a^
	No	29 (44.6)	45 (69.2)	
Medication for the pain relief	Yes	50 (76.9)	34 (52.3)	0.003 ^a^
	No	15 (23.1)	31 (47.7)	
Treatment with insulin	Yes	45 (69.2)	33 (50.8)	0.032 ^a^
	No	20 (30.8)	32 (49.2)	
Physical comorbidity	Yes	59 (90.8)	59 (90.8)	1 ^a^
	No	6 (9.2)	6 (9.2)	
HADS Anxiety total	Mean (SD)	9.29 (5.36)	4.29 (4.25)	0.000 ^d^
HADS Depression score	Mean (SD)	8.74 (5.4)	4.82 (3.86)	0.000 ^d^
Previous history of anxiety	Yes	24 (36.9)	6 (9.2)	0.000 ^a^
	No	41 (63.1)	59 (90.8)	
Previous history of depression	Yes	26 (40)	8 (12.3)	0.000 ^a^
	No	39(60)	57 (87.7)	
US Standardized physical component	Mean (SD)	30.35 (10.25)	38.92 (11.81)	0.000 ^c^
US Standardized mental component	Mean (SD)	42.1 (14.09)	52.95 (11.62)	0.000 ^d^
**Associated complications**				
Diabetic retinopathy	Yes	23 (35.4)	20 (30.8)	0.576 ^a^
	No	42 (64.6)	45 (69.2)	
Diabetic nephropathy	Yes	18 (27.7)	23 (35.4)	0.345 ^a^
	No	47 (72.3)	42 (64.6)	
Diabetic foot	Yes	21 (32.3)	19 (29.2)	0.704 ^a^
	No	44 (67.7)	46 (70.8)	
Cardiovascular disease	Yes	30 (46.2)	28 (43.1)	0.724 ^a^
	No	35 (53.8)	37 (56.9)	
**Cardiovascular risk factors**				
Obesity	Yes	41 (63.1)	25 (38.5)	0.005 ^a^
	No	24 (36.9)	40 (61.5)	
Arterial hypertension	Yes	48 (73.8)	53 (81.5)	0.292 ^a^
	No	17 (26.2)	12 (18.5)	
Dyslipidemia	Yes	52 (80)	41 (63.1)	0.033 ^a^
	No	13 (20)	24 (36.9)	

^a^ Pearson’s chi-squared; ^b^ Likelihood ratio; ^c^ Student T; ^d^ Mann–Whitney U.

**Table 2:** Medical Outcomes Study (MOS) sleep dimensions scores in DNP patients and no DNP patients.

Sleep Dimensions	Measure	Cases (Yes Pain DN4 ≥ 4) *n* = 65	Controls (No Pain DN4 ≤ 4) *n* = 65	*p*-Value
Sleep disturbance ^a^	Mean (SD)	54.94 (28.4)	34.13 (25.2)	<0.001 ^d^
Daytime somnolence ^a^	Mean (SD)	49.03 (24.5)	38.26 (23.2)	0.014 ^d^
Sleep adequacy ^a^	Mean (SD)	57.23 (35.4)	28.92 (27.8)	<0.001 ^d^
Snoring ^a^	Mean (SD)	53.85 (36.9)	44.92 (37.3)	0.160 ^d^
Short of breath or headache ^a^	Mean (SD)	27.69 (29.9)	12.62 (20.5)	0.003 ^d^
Sleep quantity (hours/night) ^b^	Mean (SD)	5.76 (1.9)	6.49 (1.7)	0.025 ^d^
Sleep problems Index (I-9) ^a^	Mean (SD)	48.79 (23.9)	28.57 (19.7)	<0.001 ^d^
Optimal sleep ^c^	%	20%	36.9%	0.033 ^e^

MOS: Medical Outcomes Study; DNP: Diabetic Neuropathic Pain. ^a^ Higher scores indicate more of the concept being measured; ^b^ quantity of sleep scores are the patient-reported number of hours of sleep per night; ^c^ if the patients report 7 or 8 h of sleep per night. The optimal sleep score is 1; otherwise the optimal score is 0; ^d^ Mann–Whitney U test; Pearson’s chi-squared test.

**Table 3:** Correlation between MOS sleep dimensions scores and Neuropathic Pain Symptom (NPSI) dimensions in DNP patients.

Sleep Dimensions		Evoked Pain	Deep Spontaneous Pain	Superficial Spontaneous Pain	Paroxysmal Pain	Paraesthesia/Dysesthesia
**Sleep disturbance ^a^**	r ^d^	0.056	0.077	0.025	0.213	0.230
**Daytime somnolence ^a^**	r ^d^	0.172	−0.066	−0.088	0.125	−0.117
**Sleep adequacy ^a^**	r ^d^	0.290 *	0.107	0.058	0.305 *	0.188
**Snoring ^a^**	r ^d^	0.106	−0.129	0.076	−0.062	−0.076
**Short of breath or headache ^a^**	r ^d^	0.421 **	0.293 *	0.191	0.092	0.269 *
**Sleep quantity (hours/night) ^b^**	r ^d^	−0.307 *	−0.030	0.115	−0.232	−0.119
**Sleep problems Index (I-9) ^a^**	r ^d^	0.276 *	0.093	0.028	0.277 *	0.208
**Optimal sleep ^c^** **Yes** **No**	Mean (SD) ^e^	2.64 (2.2) 3.37 (2.7)	4.96 (3.8) 3.42 (3.2)	6.62 (3.6) ** 3.67 (3.8)	4.58 (2.9) 4.91 (2.9)	5.85 (2.8) 5.33 (2.7)

MOS: Medical Outcomes Study; NPSI: Neuropathic Pain Symptom Inventory; DNP: Diabetic Neuropathic Pain. ^a^ Higher scores indicate more of the concept being measured; ^b^ Quantity of sleep scores are the patient-reported number of hours of sleep per night; ^c^ If the patients report 7 or 8 h of sleep per night. the optimal sleep score is 1; otherwise the optimal score is 0; ^d^ r: Spearman Correlation Coefficient; ^e^ Mann–Whitney U test; * *p* < 0.05; ** *p* < 0.01.

**Table 4:** Factors related to sleep quality (Sleep Problems Index) in DNP patients. Bivariate analysis.

Variables	Categories	MOS I- 9 Index (*n =* 65)		*p*-Value
		Nº	Mean (SD)	
**Sociodemographic data**				
Sex	Men Women	27 38	43.48 (23.02) 52.57 (24.16)	0.126 ^a^
Age	Correlation coefficient: −0.204			0.103 ^b^
Educational level	No education Primary studies Secondary studies University studies	20 33 10 2	54.72 (23.22) 50.47 (23.83) 37.17 (22.15) 20 (6.28)	0.087 ^c^
Employment status	Unemployed Homemaker Working Retired Partial disability Total disability	5 23 3 22 1 11	68 (26.92) 53.53 (22.67) 37.41 (13.34) 42.05 (24.27) 47.17 (24.39) 48.79 (23.94)	0.206 ^c^
**Clinical data**				
Evolution time of DNP (months)	Correlation coefficient: 0.039			0.761 ^b^
Intensity of the DNP (VAS) (0–10)	Correlation coefficient: 0.31			0.012 ^b^
Time since type-2 diabetes mellitus diagnosis (years)	Correlation coefficient: −0.242			0.052 ^b^
HbA1c (*n*: 65)	Correlation coefficient: −0.284			0.022 ^b^
Medication for sleep	Yes No	36 29	54.57 (22.81) 41.63 (23.75)	0.03 ^a^
Medication for pain relief	Yes No	50 15	48.44 (24.57) 49.96 (22.48)	0.791 ^a^
Treatment with insulin	Yes No	45 20	45.12 (23.61) 57.06 (23.15)	0.067 ^a^
Physical comorbidity(*n =* 65)	Yes No	59 6	50.16 (24.34) 35.37 (15.05)	0.134 ^a^
HADS Anxiety total	Correlation coefficient: 0.613			0.000 ^b^
HADS Depression total	Correlation coefficient: 0.668			0.000 ^b^
Previous history of anxiety	Yes No	24 41	62.75 (16.17) 40.62 (24.11)	0.000 ^a^
Previous history of depression	Yes No	26 39	63.06 (16.93) 39.29 (23.34)	0.000 ^a^
US standardized physical component	Correlation coefficient: −0.172			0.171 ^b^
US standardized mental component	Correlation coefficient: −0.666			0.000 ^b^
**Associated complications**				
Diabetic retinopathy	Yes No	23 42	47.25 (22.43) 49.64 (24.95)	0.675 ^a^
Diabetic nephropathy	Yes No	18 47	52.31 (23.71) 47.45 (24.14)	0.504 ^a^
Diabetic foot	Yes No	21 44	47.62 (23.16) 49.36 (24.55)	0.731 ^a^
Cardiovascular disease	Yes No	30 35	52.8 (22.27) 45.37 (25.09)	0.286 ^a^
**Cardiovascular risk factors**				
Obesity	Yes No	41 24	50.58 (25.6) 45.74 (20.97)	0.442 ^a^
Arterial hypertension	Yes No	48 17	50.36 (24.33) 44.38 (22.93)	0.386 ^a^
Dyslipidemia	Yes No	52 13	49.66 (24.74) 45.34 (20.95)	0.599 ^a^

DNP: Diabetic Neuropathic Pain; I-9 Index: Sleep Problem Index. HADS: Hospital Anxiety and Depression scale. ^a^ Mann–Whitney U; ^b^ Spearman’s correlation; ^c^ Kruskal-Wallis.

**Table 5:** Multiple linear regression models of the factors related with the sleep quality (Sleep Problem Index (I-9)) in DNP patients (*n* = 65).

Variables	Coefficients (SE)	CI (95%)	*p*-Value
**Model 1 (R^2^ = 0.545)**			
Constant	52.74 (15.3)	(22.04; 83.44)	0.001
Intensity of the DNP (VAS)	2.19 (1.0)	(0.21; 4.16)	0.031
Time since type-2 diabetes mellitus diagnosis (years)	−1.34 (0.7)	(−2.65; −0.04)	0.044
HbA1c	−3.99 (1.5)	(−6.96; −1.03)	0.009
HADS Anxiety total	1.35 (0.54)	(0.27; 2.42)	0.015
HADS Depression total	1.46 (0.52)	(0.42; 2.49)	0.007
**Model 2 (R^2^ = 0.563)**			
Constant	60.1 (14.33)	(31.42; 88.77)	<0.001
Paroxysmal pain	1.93 (0.71)	(0.52; 3.35)	0.008
Time since type-2 diabetes mellitus diagnosis (years)	−1.13 (0.63)	(−2.38; 0.13)	0.077
HbA1c	−4.49 (1.46)	(−7.41; −1.58)	0.003
HADS Anxiety total	1.09 (0.54)	(0.01; 2.18)	0.048
HADS Depression total	1.83 (0.51)	(0.81; 2.84)	0.001

Dependent variable: Sleep Problems Index (I−9). DNP: Diabetic Neuropathic Pain; CI: Confidence Interval.
